# Kinship as a double-edged sword: relatedness among burying beetle larvae enhances growth but increases mortality

**DOI:** 10.1098/rsbl.2025.0319

**Published:** 2025-09-10

**Authors:** Paul Huber, Daniel Wittmann, Sandra Steiger

**Affiliations:** ^1^ Evolutionary Animal Ecology, University of Bayreuth, Bayreuth, Germany

**Keywords:** cooperation, family life, kinship, social evolution, sibling interactions

## Abstract

Theoretical and empirical considerations suggest that relatedness can have complex effects on social life. While high relatedness may promote sibling cooperation and altruism through indirect fitness benefits, it can also intensify competition if siblings share similar needs and competitive strategies. Moreover, low genetic diversity in highly related groups may heighten susceptibility to pathogens. Hence, due to these potential opposing effects, the consequences of relatedness for offspring fitness within a family context are not fully understood. Here, we investigated how relatedness among interacting offspring influences their fitness in the burying beetle *Nicrophorus vespilloides*, a species exhibiting facultative parental care, with larvae developing in a microbially rich and challenging environment. To assess offspring effects without parental influence, we raised larvae in the absence of care, thereby eliminating parental buffering and exposing them to a more stressful environment. We compared the growth and survival rates of broods consisting of full siblings and broods with unrelated larvae and found both benefits and costs of relatedness. Larvae gained weight more rapidly in the early stages when surrounded by siblings but suffered higher mortality later in development. These findings suggest that high relatedness facilitates cooperative effects but comes at a cost, potentially reducing social immunocompetence.

## 1. Introduction

Kin selection theory predicts that relatedness reduces competition and promotes cooperation among individuals [1–4]. However, when interacting individuals have similar needs and competitive strategies, high relatedness can also intensify competition [5,6]. In line with this, the level of kinship within family groups is often lower than anticipated, and even in eusocial insects relatedness is often lower than expected from kin selection theory alone [7–10]. This pattern is thought to reflect the adaptive value of increased genetic diversity, driven by traits such as multiple mating and paternity concealment [5,11–13]. Through social heterosis—the synergistic benefits of interactions among genetically dissimilar individuals [14,15]—genetic diversity is proposed to reduce competition and enhance overall performance, including survival and disease resistance [14–21].

Surprisingly, while many studies have examined the effects of kinship and social heterosis in various eusocial Hymenoptera [7,8,15,17,22–25], the balance between the benefits and costs of interacting with close relatives has received little attention in less complex social systems, such as family groups consisting of parents and their offspring. Nevertheless, these dynamics are particularly relevant in family groups, where individuals live in close contact and often compete for limited resources [26]. Such conditions can promote selfish or even cheating behaviours during development. Yet, when family members are closely related, selfish behaviour may reduce indirect fitness gains, ultimately selecting against harmful strategies [1,2,27,28]. For example, relatedness has been shown to reduce begging competition in passerine birds [29], and decrease siblicidal behaviour in earwig nymphs [30,31]. Moreover, kin selection theory also predicts that relatedness should promote cooperation—a pattern supported by observations in earwigs, social spiders and juvenile cichlids, which show increased food sharing, cooperative feeding or improved growth in the presence of kin [32–36]. Building on such findings, recent research has proposed that sibling cooperation may have played a key role in the early evolution of social and family life [37]. Not only in highly social species, but even in those with facultative family life, offspring have been shown to benefit from growing up alongside siblings [32,38–43]. On the other hand, some evidence suggests similar benefits of social heterosis in family groups: brown trout grow larger [44], and pygmy grasshoppers show increased survival in mixed-parentage broods [45]. Following the contrasting observations, it remains unclear how variation in relatedness and genetic diversity affects cooperative behaviour and the benefits of sibling interactions in the context of early social and family life.

Here, we took advantage of a convenient model organism for the study of the early evolution of family life, the burying beetle *Nicrophorus vespilloides*. With their temporary and facultative family structure—ranging from no care to uni- and biparental care, and even cooperative breeding—burying beetles represent an early form of social organization [37] and offer valuable insights into the origins of more complex social systems. Since burying beetles reproduce on small vertebrate carcasses, their offspring develop in an environment that is both highly competitive and pathogen rich [46]. The carcass serves as a feeding resource for both parents and offspring. In the absence of parents, *N. vespilloides* larvae show faster growth and achieve higher larval masses—traits correlated with adult body size—when raised in larger broods [39,42,47], indicating that larvae may benefit from cooperative behaviours during development. While the exact mechanisms underlying this cooperation are not yet fully understood, several possibilities have been proposed, including pooling of exodigestive enzymes, collective feeding behaviour and thermal advantages [39,41,42]. As females mate multiple times before oviposition and brood parasitism is common, broods are often of mixed parentage [48,49]. However, how relatedness affects offspring interactions, especially under harsh environmental conditions, has not yet been tested. Here, we therefore measured how relatedness affects offspring performance (larval growth, body mass and brood survival) in the absence of parental care. We compared the performance of *N. vespilloides* larvae in two treatments—full-sibling broods and broods of unrelated individuals—using standardized and equal brood sizes across treatments. Based on theoretical considerations and previous studies, we expected that relatedness could influence offspring performance in multiple ways. It may promote cooperation and enhance early growth, but it could also increase competition among similarly behaving individuals, potentially reducing growth. Moreover, the associated reduction in genetic diversity may impair social immune competence [8,20], leading to higher mortality later in development as microbial threats accumulate.

## 2. Material and methods

### (a) Study animals

The experimental beetles were laboratory-bred descendants of individuals caught in the Studentenwald in Bayreuth, Germany (49°55′5.18″ N, 11°34′58.88″ E). In the lab, beetles were housed in plastic boxes (10 × 10 × 6 cm), separated by sex and family, with half of the box filled with moist coco peat. They were kept in a climate chamber at 20°C under a 16 : 8 h light : dark cycle. To breed beetles, unrelated virgin male and female pairs were given access to 20 g mouse carcasses.

### (b) Experimental procedure

To test whether relatedness within a brood affects larval growth and survival, we established two treatment groups: a full-sibling treatment, consisting of a standardized brood size of eight full siblings; and a non-sibling treatment, consisting of eight unrelated larvae. We deliberately chose a brood size of eight larvae based on previous research [47], which identified this number as appropriate for studying cooperative interactions among larvae without inducing food limitation late in development under comparable experimental conditions. To this end, we set up 49 pairs of unrelated female and male beetles, 19 of which were later randomly designated to produce full-sibling broods, while the remaining 30 pairs were used to produce unrelated offspring for the non-sibling treatment. Beetle pairs were set up in transparent plastic boxes (10 × 10 × 6 cm), half filled with moist coco peat, given access to a 9 g mouse carcass (mean = 8.79, s.d. = 0.81) and maintained in darkness at a constant temperature of 20°C. After 48 h, parents were removed, and carcasses were transferred to a new container. To exclude maternal effects, carcasses were mixed across the samples ensuring that no full-sibling sample received a prepared carcass from its own parents. Furthermore, an artificial hole was cut into each carcass to create a standardized feeding cavity, providing larvae with consistent access points to the nutrient-rich tissue across all samples [39,50]. At the expected time of larval hatching, we started to check for hatching every hour, day and night. For each full-sibling treatment, larvae that hatched within the same hour were weighed and added to the carcass until each brood contained eight larvae. *N. vespilloides* females do not lay all their eggs at once leading to asynchronous larval hatching [51,52]. Since the hatching spread can influence larval growth and survival [15], each sibling brood was paired with a corresponding non-sibling brood in which the hatching spread was closely replicated. To achieve this, for each non-sibling brood, each hour larvae were randomly selected from the 30 non-sibling samples in the same numbers as in the corresponding sibling brood, weighed and added to the carcass. Care was taken to ensure that no larva was related to any other larva within a non-sibling brood. Out of the 19 full-sibling samples, 15 produced at least eight larvae, resulting in overall 15 full-sibling broods and a corresponding 15 samples of non-sibling broods with a matching hatching spread. In our experiment, the hatching spread, calculated based on the first eight larvae to hatch, ranged from 1 to 5 h (mean = 2.2, s.d. = 0.92). Due to natural variation in the onset of egg laying, asymmetric hatching patterns, and the need to maintain hourly monitoring, later-hatching clutches could not be considered. After setting up the standardized broods, they were transferred back to the 20°C climate chamber. Larvae were weighed and their survival was quantified at 24 and 48 h after the first larva was added to each sample, as well as at dispersal (defined here as the time when all larvae in a brood had left the carcass). The average larval mass was calculated by dividing the total brood mass by the number of larvae. Finally, larval growth rate was calculated by subtracting the average larval hatching mass from the average larval mass at 24 h, 48 h or at dispersal, and then dividing by the average larval hatching mass ((*X*_24h/48h/disp_ − *X*_0_)/*X*_0_, with *X* being the average larval mass within a brood at the respective time point).

### (c) Statistical analysis

All statistical analyses were conducted in R (v. 4.4.3) and R-Studio (v. 2024.12.1 Build 563) with the following packages: car [53], DHARMa [54], lme4 [55], ggplot2 [56], gridExtra [57] and dplyr [58]. General mixed models (LMM) were used to test the effect of relatedness and hatching spread on the average larval mass and growth rates at 24 h, 48 h and at dispersal (all LMMs with a Gaussian error structure). Generalized linear mixed models (GLMMs with a binomial error structure) were used to test the effect of relatedness and hatching spread on the survival rates. A random effect for brood identity was included in all (G)LMMs, with the same ID assigned to each paired full-sibling and non-sibling brood that shared the same hatching spread. Residuals of all models were tested for normality, over- or under-dispersion and variance homogeneity using the DHARMa package. In addition, a Wilcoxon rank sum test was used to compare the time of dispersal between the sibling and non-sibling groups.

## 3. Results

**Figure 1.**
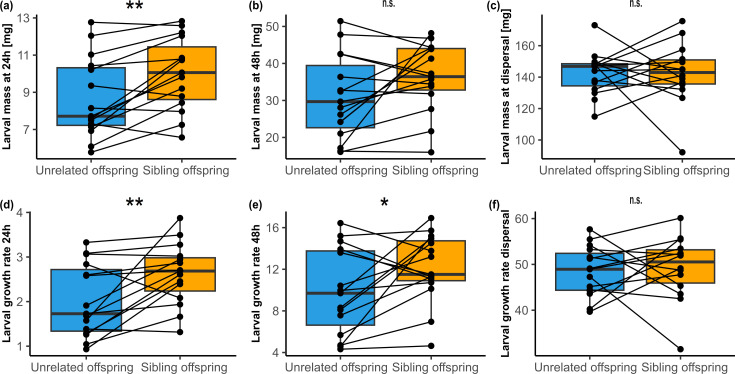
Average larval masses (a–c) and growth rates (d–f) of offspring growing alongside unrelated larvae or full siblings at 24 h (a,d), 48 h (b,e) and dispersal (c,f). Lines connect each full-sibling brood to its corresponding non-sibling brood, in which the hatching spread was replicated using unrelated larvae. Box plots illustrate median and interquartile range, with whiskers extending to 1.5 times the interquartile range. Stars represent significant differences with *p < 0.05, **p < 0.01 and ***p < 0.001.

**Figure 2.**
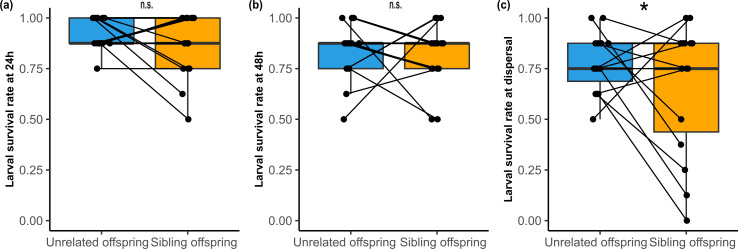
Survival rates of unrelated offspring and full siblings at 24 h (a), 48 h (b) and dispersal (c). Points represent individual brood survival rates, with lines connecting the corresponding sibling and non-sibling samples. Box plots illustrate median and interquartile range, with whiskers extending to 1.5 times the interquartile range. Stars represent significant differences with *p < 0.05, **p < 0.01 and ***p < 0.001.

While there was no difference in larval mass at hatching (*F*_1,14_ = 1.64, *p* = 0.22; electronic supplementary material, figure S1a), larvae growing alongside kin were larger than those growing alongside unrelated larvae at 24 h (*F*_1,14_ = 13.69, *p* = 0.002; figure 1a and electronic supplementary material, figure S1a), resulting in a higher associated growth rate (*F*_1,14_ = 9.02, *p* = 0.009; figure 1d and electronic supplementary material, figure S1b). This difference cannot be attributed to differences in larval number, as survival rate did not differ between the treatments (*χ*^2^_1_ = 1.55, *p* = 0.21; figure 2a and electronic supplementary material, figure S1c). Neither larval mass nor growth rate at 24 h was affected by hatching spread (both *p* > 0.05), but survival decreased with increasing hatching spread (*χ*^2^_1_ = 3.88; *p* = 0.049).

At 48 h, larval mass tended to be higher in sibling broods than in non-sibling broods (*F*_1,14_ = 3.69, *p* = 0.08; figure 1b and electronic supplementary material, figure S1a), resulting in a higher growth rate in sibling larvae (*F*_1,14_ = 4.50, *p* = 0.05; figure 1d and electronic supplementary material, figure S1b). Again, no difference in the survival rate between treatments was found (*χ*^2^_1_ = 0.12, *p* = 0.73; figure 2b and electronic supplementary material, figure S1c). At this time point, larval mass, growth rate and survival were not affected by hatching spread (all *p* > 0.05).

At dispersal, neither larval mass (*F*_1,13.72_ < 0.001, *p* = 0.99; figure 1c and electronic supplementary material, figure S1a) nor growth rate (*F*_1,13.69_ = 0.13, *p* = 0.72; figure 1f and electronic supplementary material, figure S1b) differed between the two treatments, but unrelated offspring had a higher survival rate than sibling offspring (*χ*^2^_1_ = 5.16, *p* = 0.02; figure 2c and electronic supplementary material, figure S1c). There was no difference in the dispersal time of sibling and non-sibling broods (*W* = 141, *p* = 0.12). Furthermore, the hatching spread influenced larval growth rate (*F*_1,12.62_ = 4.83; *p* = 0.047), but not larval mass (*F*_1,12.62_ = 1.28, *p* = 0.28) or survival (*χ*^2^_1_ = 0.99, *p* = 0.32) at dispersal.

## 4. Discussion

While relatedness is expected to increase cooperation [1,2], it can also increase resource competition and decrease immune competence [5,16,17]. Here, we used the subsocial burying beetle *N. vespilloides* to examine these contrasting effects of relatedness on offspring growth and survival. We found that related offspring grew faster but had a higher mortality rate than unrelated offspring. This supports the idea that there is indeed simultaneous but conflicting selection for and against high relatedness in early family groups.

Sibling presence is known to have beneficial effects in burying beetles. For instance, sibling presence can mitigate the costs of parental absence, and larvae in larger broods feature higher growth rates [39,42,47], even in mixed parentage broods [39,41,42,47]. Thus, larvae growing in larger broods spent less time in the most vulnerable ontogenic period [59,60] and in a highly infectious and competitive environment [46]. Our results show that relatedness further increases early growth rates, suggesting that kinship enhances these benefits of sibling interactions in the absence of parental care. However, the mechanism underlying the positive effect of sibling presence remains unclear. Previous studies suggest that larvae may benefit from aggregation by gaining more efficient access to carcass tissue through collective feeding efforts, by pooling exodigestive enzymes, or by creating thermal advantages [39,41,42]. The observed positive effect of relatedness on early growth may result from active kin discrimination, with larvae adjusting their cooperative behaviour depending on kinship. Kin-directed cooperative behaviours have been documented in a range of taxa, including some insect species and their offspring. For example, earwig nymphs adjust food-sharing behaviour in the presence of kin [32], and nymphs of the German cockroach *Blattella germanica* form sibling-based subgroups in large aggregates, thereby sharing direct and indirect fitness benefits with closely related individuals [61]. It is possible that, similar to cockroach nymphs, *Nicrophorus* larvae exhibit a preference for aggregating more closely with kin on the carrion resource. Larvae may also adjust digestive enzyme production based on the relatedness of their group members. However, such behaviours would imply an ability for kin discrimination. While *Nicrophorus* adults appear capable of distinguishing between their own and foreign eggs [62], and *N. vespilloides* larvae can discriminate between familiar and unfamiliar breeding adults [63] and possess a complex chemical profile that may allow chemically mediated kin recognition [64] direct evidence for kin discrimination among larvae remains lacking and cannot be inferred from our study. In fact, our findings may be explained by mechanisms that do not require kin discrimination: larvae with similar genotypes may behave more similarly, which could facilitate coordination and increase the efficiency of collective actions. These synergistic interactions could improve access to carcass nutrients and accelerate growth.

In contrast to its positive effects on early growth, we found that high relatedness reduced the survival of burying beetle larvae in the long run, a pattern that may appear counterintuitive at first glance. The increased mortality is unlikely to be the result of siblicide. Although parental infanticide is common in burying beetles [65–67], siblicidal behaviour—unlike in other insect species [30,31]—has not been observed in *Nicrophorus* larvae. Starvation also seems unlikely. Sufficient food was provided to support eight larvae through development, and mortality occurred later in the cycle, when larvae are efficient in self-feeding [50,68]. Moreover, since sibling larvae were larger in the early phase, starvation is an unlikely explanation for their higher mortality [46]. A more likely explanation is that relatedness affects the brood’s capacity to combat pathogenic microbes, which become increasingly problematic later in development due to microbial growth and toxin accumulation [69–72]. Parental care involves treating the carcass with antimicrobial secretions that reduce microbial growth [46,70,72,73]. These secretions contain antimicrobial peptides (AMPs) and different lysozymes [74–76]. Larvae also invest in social immunity by producing antimicrobial substances of their own [77–79]. In genetically diverse broods, pooled larval secretions may include a wide range of AMPs, which can act synergistically or in complementary ways [80], potentially enhancing social immune competence and reducing pathogen impact.

This idea aligns with broader evidence that genetic diversity supports pathogen resistance. For example, strong individual and population differences in AMP expression were found in bumble bees (*Bombus terrestris*) [81,82], which have been shown to act synergistically against pathogens [83]. In line with this, an artificial increase in polyandry to increase genetic diversity enhances parasite resistance in this species [17]. Similarly, in freshwater snails *Potamopyrgus antipodarum*, pathogen exposure leads females to increase the number of mating partners, which have been suggested to enhance genetic diversity and immune competence of the offspring [13]. Moreover, a meta study showed that while kinship is beneficial in pathogen-free environments, in the presence of pathogens, genetic diversity can outweigh the benefits of kinship [20]. Notably, previous studies in *N. vespilloides* found no clear fitness effects of multiple mating [84,85], likely because they were conducted in the presence of parental care, which buffers against environmental stress and can mask potential benefits or costs of genetic diversity. To determine whether reduced social immune competence is indeed responsible for the lower survival of related larvae or if other factors play a role, we encourage future experiments under more severe microbial challenges, where the effect of social immune competence should be even stronger if it is a key factor.

While benefits associated with genetic diversity for the offspring under harsh environmental conditions could not only be a potential explanation for multiple matings by *Nicrophorus* females, they could also be a driver for the evolution and maintenance of cooperative breeding by multiple *Nicrophorus* females or pairs [86,87]. In our study, we created an experimental extreme by assembling non-sibling broods in which no individuals shared a parent. Although such a scenario is unlikely in nature, it is commonly used in experimental designs where larvae are pooled and redistributed across treatments to reduce genetic effects, standardize brood size and/or control for variation in hatching spread [38,41,42]. Our findings suggest that this practice may unintentionally bias estimates of larval performance, at least under harsh environmental conditions, such as when larvae develop without their parents. At the same time, it is clear that natural broods in *Nicrophorus* are often not composed entirely of full siblings. Multiple mating, sperm storage, brood parasitism, satellite males and cooperative breeding can all contribute to mixed relatedness within broods [49]. However, since the actual distribution of relatedness in wild broods remains poorly understood, more empirical data are needed to assess how naturally occurring kin structure shapes larval interactions. It would also be valuable to examine how intermediate levels of relatedness, such as among half-siblings, influence larval performance.

Taken together, our results point to an evolutionary trade-off in the benefits of low versus high genetic relatedness in family groups. While high genetic relatedness can promote cooperation through direct and indirect fitness benefits, low relatedness may enhance group-level fitness through social heterosis, particularly by improving immune competence. This tension may generate selection for the evolution of kin recognition. At the same time, the benefits of genetic diversity could favour the evolution of multiple matings, mixed parentage broods and paternity concealment. Thus, the balance between relatedness-driven cooperation and diversity-driven resilience may shape both social behaviour and mating strategies in family-living species and may underlie the diversity of family forms observed in nature.

## Data Availability

Data and R code are available from the Zenodo Digital Repository [88]. Supplementary material is available online [89].
